# Increased diversity with reduced “diversity evenness” of tumor infiltrating T-cells for the successful cancer immunotherapy

**DOI:** 10.1038/s41598-018-19548-y

**Published:** 2018-01-18

**Authors:** Akihiro Hosoi, Kazuyoshi Takeda, Koji Nagaoka, Tamaki Iino, Hirokazu Matsushita, Satoshi Ueha, Shin Aoki, Kouji Matsushima, Masato Kubo, Teppei Morikawa, Kazutaka Kitaura, Ryuji Suzuki, Kazuhiro Kakimi

**Affiliations:** 1 Department of Immunotherapeutics, The University of Tokyo Hospital, 7-3-1 Hongo, Bunkyo-Ku, Tokyo, 113-8655 Japan; 2 Division of Cell Biology, Biomedical Research Center, Graduate School of Medicine, Juntendo University, Bunkyo-ku, Tokyo, 113-8421 Japan; 3 Department of Biofunctional Microbiota, Graduate School of Medicine, Juntendo University, Bunkyo-ku, Tokyo, 113-8421 Japan; 4 Department of Molecular Preventive Medicine, Graduate School of Medicine, The University of Tokyo, 7-3-1 Hongo, Bunkyo-Ku, Tokyo, 113-0033 Japan; 5 Laboratory for Cytokine Regulation, Research Center for Integrative Medical Science (IMS), RIKEN Yokohama Institute, Yokohama, Kanagawa 230-0045 Japan; 6 Division of Molecular Pathology, Research Institute for Biomedical Science, Tokyo University of Science, Noda, Chiba 278-0022 Japan; 7 Department of Pathology, Graduate School of Medicine, The University of Tokyo, 7-3-1 Hongo, Bunkyo-Ku, Tokyo, 113-0033 Japan; 8 Repertoire Genesis Inc., Saito Bioincubator 104, 7-7-15 Saito-asagi, Ibaraki-shi, Osaka, 567-0085 Japan; 9 MEDINET Co., Ltd 2-3-12 Shin-Yokohama, Kohoku-ku, Yokohama, Kanagawa 222-0033 Japan

**Keywords:** Tumour immunology, Cancer microenvironment

## Abstract

To facilitate the optimization of cancer immunotherapy lacking immune-related adverse events, we performed TCR repertoire analysis of tumor-infiltrating CD8^+^ T-cells in B16 melanoma-bearing mice receiving anti-PD-1, anti-CTLA-4, anti-4-1BB, anti-CD4 or a combination of anti-PD-1 and 4-1BB antibodies. Although CD8^+^ T-cells in the tumor were activated and expanded to a greater or lesser extent by these therapies, tumor growth suppression was achieved only by anti-PD-1, anti-PD-1/4-1BB combined, or by anti-CD4 treatment, but not by anti-CTLA-4 or anti-4-1BB monotherapy. Increased CD8^+^ T cell effector function and TCR diversity with enrichment of certain TCR clonotypes in the tumor was associated with anti-tumor effects. In contrast, polyclonal activation of T-cells in the periphery was associated with tissue damage. Thus, optimal combination therapy increases TCR diversity with extended activation of selective CD8^+^ T-cells specifically in the tumor but not in the periphery. Incorporation of the concept of evenness for the TCR diversity is proposed.

## Introduction

Immunomodulatory cancer immunotherapy using cytotoxic T lymphocyte antigen 4 (CTLA-4) or programmed cell death 1 receptor (PD-1)-specific checkpoint blockade provides substantial clinical benefits for a minority of cancer patients by unleashing their own anti-tumor immunity^1,2^. These blocking antibodies inhibit the interaction of CTLA-4 or PD-1 receptors on T-cells with their ligands on tumor cells or antigen-presenting cells and can reinvigorate tumor-reactive T-cells that have become dysfunctional or exhausted in the immunosuppressive tumor microenvironment^3,4^. However, the proportion of patients benefiting from these therapies is limited^5^, emphasizing the need to identify which patients will respond to immunotherapies and to determine reasons for treatment success or failure. To this end, it is likely that the development of synergistic treatment combinations based on immune checkpoint blockade will be required.

To gain mechanistic insights for designing more effective combination immunotherapies, we utilized the challenging B16 murine melanoma model to investigate the nature of the intratumoral immune response induced by checkpoint blockade with anti-PD-1 or anti-CTLA-4 monoclonal antibodies (mAbs), or immunostimulatory anti-4-1BB antibody^6^ or anti-CD4 mAb which can deplete immunosuppressive leukocyte populations^7^. Here, we examined (i) anti-tumor effects by measuring suppression of tumor growth, (ii) the degree of T-cell expansion and infiltration into the tumor, (iii) T-cells’ antigen experience and IFNγ production, (iv) TCR diversity. We have integrated this information regarding TCR repertoire, T-cell functions and anti-tumor activities and examined associated immune-related adverse events. Our results should contribute to a better understanding of the role of tumor-infiltrating T lymphocytes in immunotherapy and allow us to develop more effective combination treatments with less immune-related adverse events.

## Results

### Anti-tumor activities of immunomodulatory antibodies

**Figure 1:**
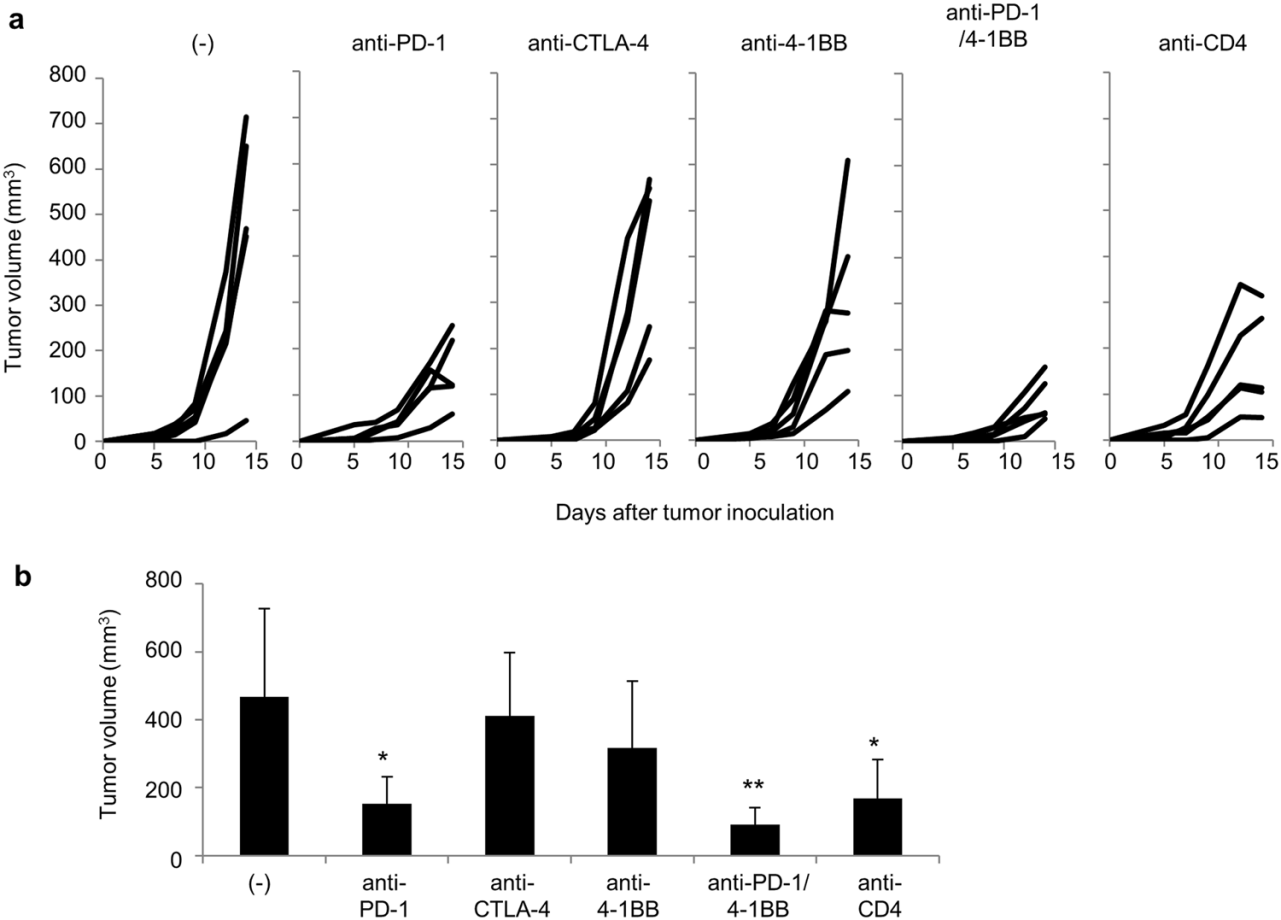
*In vivo* anti-tumor activity of cancer immunotherapies. (**a**) IFNγ Venus mice (5 mice per group) were subcutaneously injected with B16 melanoma cells (5 × 10^5^). Tumor volumes were measured every other day. Mice were untreated or given 200 μg of monoclonal antibodies against PD-1, CTLA-4, 4-1BB, CD4 or the combination of anti-PD-1 and anti-4-1BB (anti-PD-1/4-1BB) on days 5 and 9. The graphs show tumor volume of individual mice. (**b**) Tumor volumes at day 14 were compared. Data are representative of two experiments with 5 mice per group. Dunnett’s test was used for multiple comparisons between control and treatment groups.*p < 0.05, **p < 0.01.

To investigate the quality and quantity of anti-tumor immune responses in the tumor and their correlation with the success or failure of cancer immunotherapy, we treated IFNγ-venus reporter mice bearing the B16 melanoma with different immunomodulatory antibodies. Mice (5 per group) first received a subcutaneous inoculation of B16F10 melanoma cells (5 × 10^5^). On days 5 and 9 they were given 200 μg of mAbs against either PD-1, CTLA-4, 4-1BB, or CD4 or a combination of anti-PD-1 and anti-4-1BB (anti-PD-1/4-1BB). As shown in Fig. 1, tumors grew progressively in untreated mice, but anti-PD-1 mAb treatment inhibited tumor growth, while anti-CTLA-4 had no apparent effect in this model. Although no marked anti-tumor activity was observed using the immunostimulatory anti-4-1BB mAb as a single agent, the combination of anti-PD-1 with 4-1BB mAb inhibited tumor growth potentially more effectively than PD-1 blockade alone. As reported previously^7^, tumor growth was also significantly inhibited by anti-CD4 mAb treatment.

### Effective immunotherapy recruits CD8^+^ T-cells to the tumor

**Figure 2:**
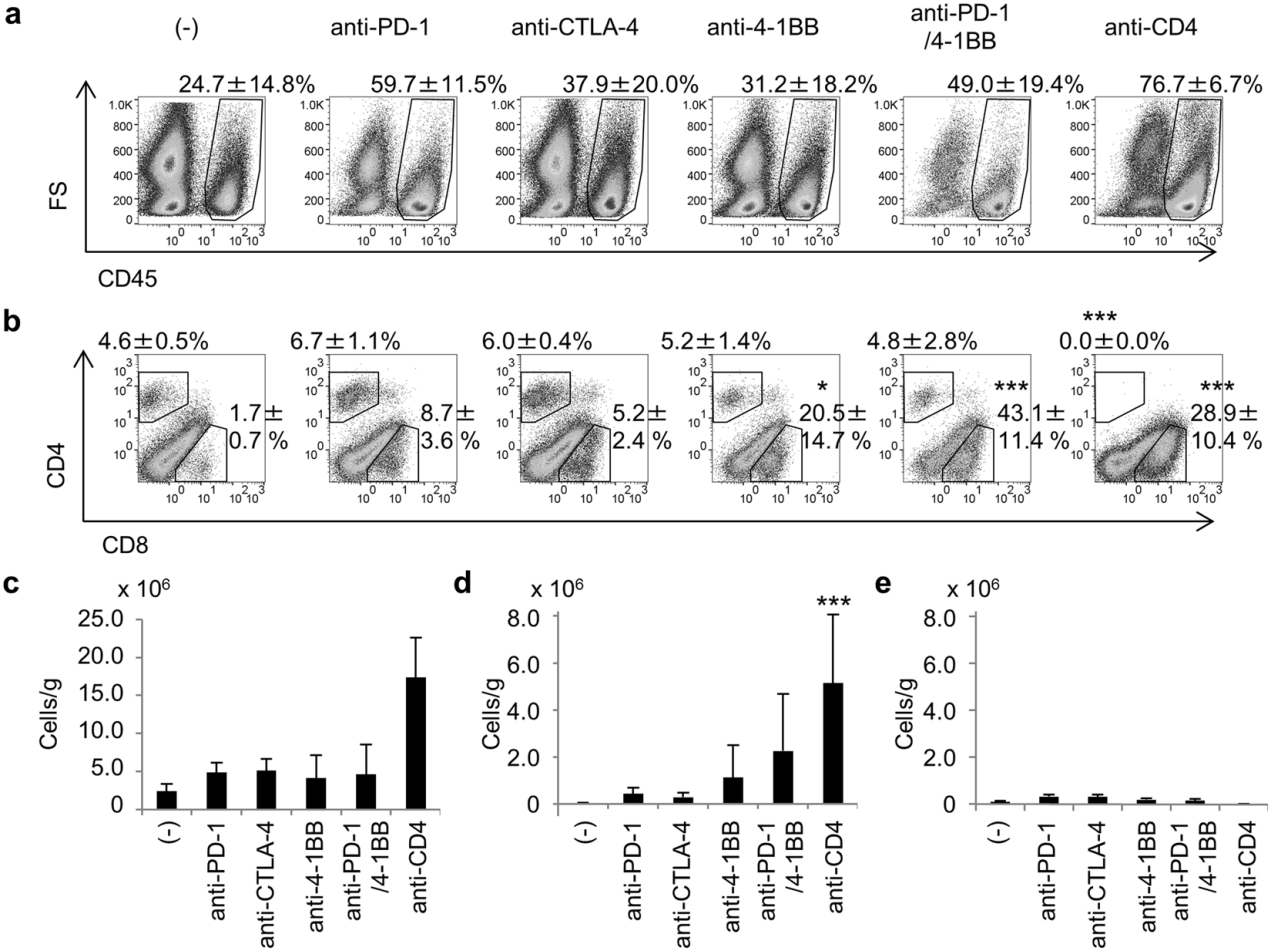
Tumor-infiltrating cells. Mice were treated as described in the legend to Fig. 1. Mice (n = 5) were killed on day 14 and tumor-infiltrating cells were analyzed by flow cytometry. (**a**) Tumor-infiltrating immune cells detected as viable cell dye Zombie Yellow^-^ CD45^+^ cells. (**b**) Percentage of CD8^+^ and CD4^+^ cells in the CD45^+^ population. The number on each panel indicates the mean ± SD of the percentage of indicated cells of 5 mice. The absolute numbers of CD45^+^ (**c**), CD8^+^ (**d**), and CD4^+^ (**e**) cells were calculated as described in the Methods section and adjusted by tumor weight (cells/g). Dunnett’s test was used for multiple comparisons between control and treatment groups (**b**,**d**). Steel’s test was used for multiple comparisons between control and treatment groups (**a**,**c**,**e**). ***p < 0.001.

All of the immunomodulatory mAb therapies tested in this study increased the infiltration of CD45^+^ immune cells into the tumor (Fig. 2a and c). These were both CD8^+^ and CD4^+^ T-cells, but more CD4^+^ than CD8^+^ T-cells were present in the tumors of untreated control mice and anti-CTLA-4-treated mice, while more CD8^+^ than CD4^+^ T-cells were found in the tumors of mice on the other treatments (Fig. 2b). The recruitment of CD8^+^ T-cells into the tumor was more marked in mice where tumor growth was suppressed by the anti-PD-1/4-1BB mAb combination or by anti-CD4 alone (Figs 1a and 2b). As expected, no CD4^+^ T-cells were detected in the tumor of anti-CD4-treated mice.

### Activation of CD8^+^ T-cells

**Figure 3:**
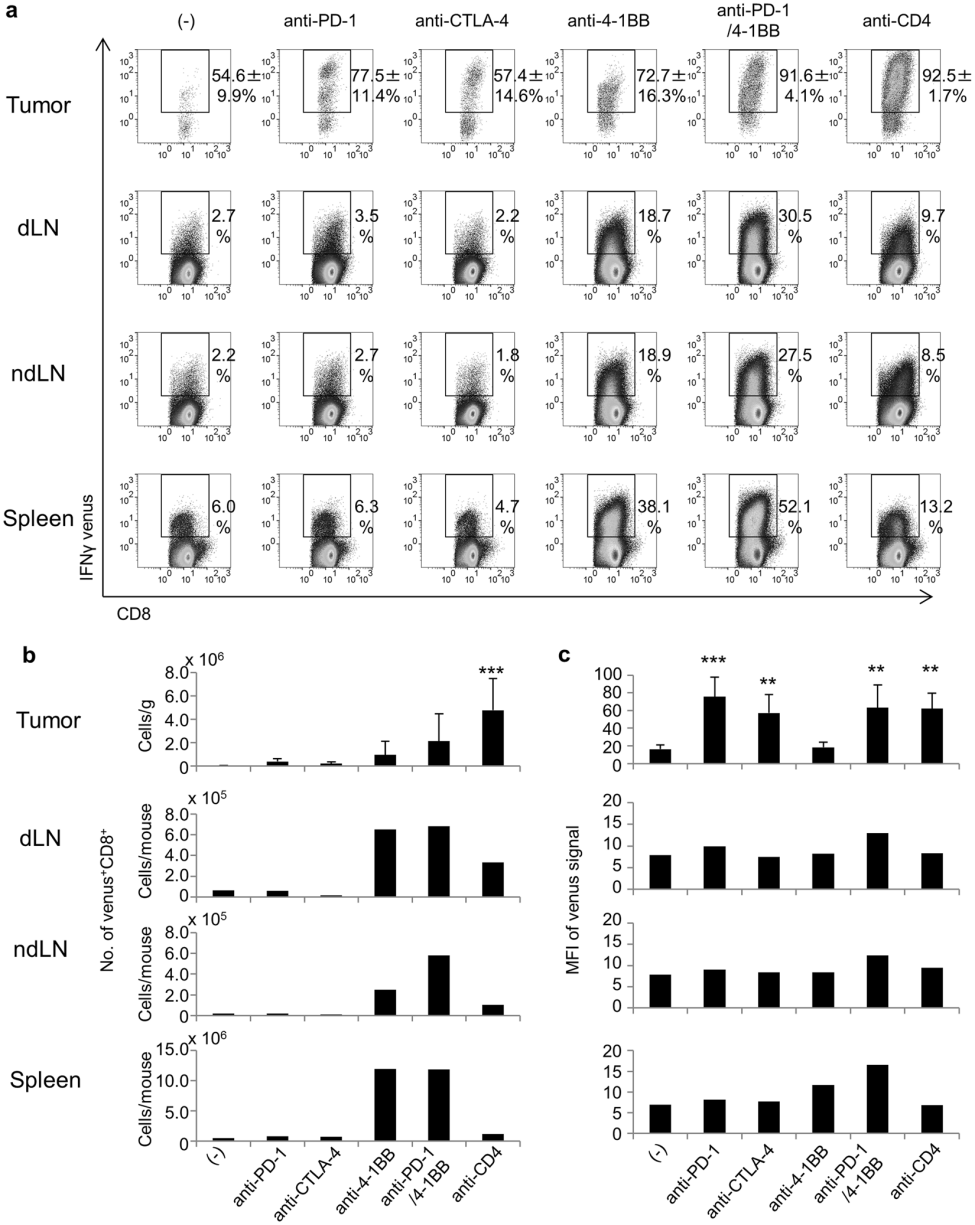
Activation of CD8^+^ T-cells by immunotherapies. (**a**) Mice were treated as described in the legend to Fig. 1. Mice (n = 5) were killed on day 14 and the IFNγ venus signal from CD8^+^ T-cells in the tumor, draining lymph node (dLN), non-draining lymph node (ndLN) and spleen was analyzed by flow cytometry. The number on each panel indicates the mean ± SD of the percentage of venus^+^ cells among CD8^+^ T-cells of 5 mice. (**b**) The absolute numbers of venus^+^ CD8^+^ T-cells and (**c**) mean fluorescent intensities (MFI) of venus signals of these cells were compared. Steel’s test was used for multiple comparisons between control and treatment groups (**a**). Dunnett’s test was used for multiple comparisons between control and treatment groups (**b**,**c**). **p < 0.01, ***p < 0.001.

To determine whether the tumor-infiltrating CD8^+^ T-cells had become activated, presumably as a result of antigenic stimulation, tumor-infiltrating lymphocytes (TILs) were isolated and analyzed by flow cytometry for the presence of the venus signal (surrogate marker for antigen recognition and IFNγ production). As shown in Fig. 3a and b, venus^+^ CD8^+^ T-cells accumulated in the tumor. Even in untreated mice, >50% of the CD8^+^ T-cells in the tumor were venus^+^ (Fig. 3a) as were >70% in mice receiving anti-PD-1 mAb or anti-4-1BB mAb. Moreover, the percentage of venus^+^ CD8^+^ was even higher (>90%) in mice treated with the anti-PD-1/4-1BB mAb combination, but also in mice treated with anti-CD4 mAb alone (Fig. 3a). In contrast, only very few venus^+^ CD8^+^ T-cells were detected in the spleen, blood, and lymph nodes (LN) of any of the mice, with the exception of those which had received anti-4-1BB mAb. These results suggest that the majority of intratumoral CD8^+^ T-cells were reactive, possibly to the tumor, but their specificity was unknown.

The mean fluorescence intensity (MFI) of venus signals was stronger in the CD8^+^ T-cells in the tumor than in the LNs or spleens (Fig. 3a and c). Interestingly, the venus signals from the intratumoral CD8^+^ T-cells exhibited two peaks of high and intermediate intensity (Fig. 3a). Venus^high^ CD8^+^ T-cells were only detected in the tumor, not in the LNs or spleen. However, CD8^+^ T-cells in mice treated only with anti-4-1BB mAb, as well as in control mice, were exclusively venus^intermediate^ (Fig. 3a). Venus^high^ CD8^+^ T-cells were detected in the tumors of mice under anti-PD-1, anti-CTLA-4, anti-CD4, or anti-PD-1/4-1BB therapy (Fig. 3a and c). These results suggest that tumor-reactive T-cells were activated and became venus^+^ as a result of the immunotherapies applied in this study, but that their activity is limited in the tumor microenvironment unless immunosuppressive mechanisms can be overcome. In mice that received anti-4-1BB or anti-PD-1/4-1BB mAb treatment, substantial numbers of venus^+^ CD8^+^ T-cells were observed in the LNs and spleens in addition to the tumor (Fig. 3b). This suggests that anti-4-1BB mAb treatment can induce systemic polyclonal activation of CD8^+^ T-cells of unknown antigen specificity.

Activation of CD4^+^ T-cells was also examined by means of the IFNγ venus signal (Supplementary Fig. S1). Venus^+^ CD4^+^ T-cells were detected in the tumor, but to a lesser extent than CD8^+^ T-cells; more venus^+^ CD4^+^ T-cells were detected in the tumors of mice treated with anti-PD-1 or anti-CTLA-4 mAb than in the other groups (Supplementary Fig. S1b). Larger numbers of venus^+^ CD4^+^ T-cells were present in the spleens and LNs of mice receiving anti-4-1BB mAb as a monotherapy or in combination with anti-PD-1 than with other treatments (Supplementary Fig. S1b). These cells also displayed higher MFI compared to the other groups (Supplementary Fig. S1c). These results are also consistent with the conclusion that anti-4-1BB mAb treatment induces broad polyclonal T-cell activation.

### Antigen-specific T-cell responses

**Figure 4:**
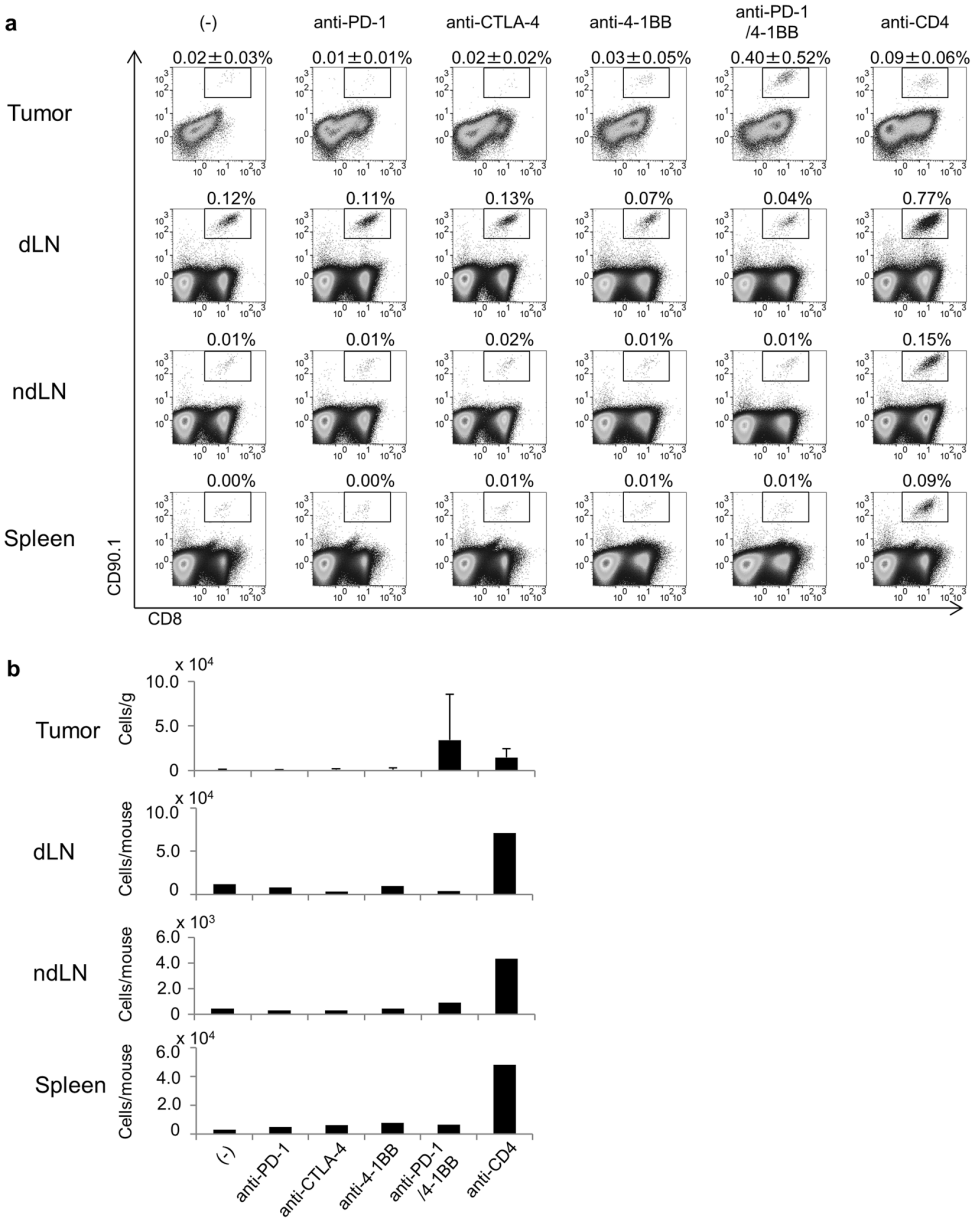
Activation of tumor-specific (gp100-specific) pmel-1 cells by immunotherapies. To increase the frequency of naive gp100-specific CD8^+^ T-cells, 5 × 10^4^ CD8^+^CD90.1^+^hgp100 tetramer^+^ cells from pmel-1 transgenic mice were adoptively transferred into IFNγ Venus mice the day before tumor challenge. Mice were then subcutaneously injected with B16 melanoma cells (5 × 10^5^). They were untreated or given 200 μg of monoclonal antibodies against PD-1, CTLA-4, 4-1BB, CD4 or the combination of anti-PD-1 and anti-4-1BB (anti-PD-1/4-1BB) on days 5 and 9. (**a**) Pmel-1 cells detected in the tumor, dLN, ndLN and spleen as CD8^+^CD90.1^+^ cells, showing the percentage of CD90.1^+^CD8^+^ T cells in CD45^+^ cells. (**b**) Absolute number of pmel-1 cells in these tissues. Steel’s test was used for multiple comparisons between control and treatment groups. (**a**,**b**).

To investigate the activation of tumor-reactive T-cells by these immunotherapies, naïve pmel-1 transgenic T-cells carrying the CD90.1 marker and known to recognize the gp100 tumor antigen were intravenously injected into C57/BL6 mice (CD90.2) the day before tumor challenge. On days 5 and 9, mice received the indicated antibodies and the expansion of pmel-1 T-cells was analyzed on day 14. Very few CD90.1^+^ pmel-1 T-cells were detected in the tumors of control mice (Fig. 4). Infiltration of pmel-1 T-cells into the tumor was seen in the anti-PD-1, CTLA-4, and 4-1BB monotherapy groups, but the degree of infiltration was similar to the controls (Fig. 4). In contrast, clear expansions of pmel-1 T-cells were seen in tumors of mice receiving the anti-PD-1/4-1BB combination, or anti-CD4 alone (Fig. 4b). More pmel-1 T-cells were detected in the draining LNs than in the non-draining LNs in all groups (Fig. 4b), suggesting that tumor-reactive T-cells were activated in the draining LNs and then infiltrated into the tumor.

### Successful immunotherapies increase the diversity of the T-cell repertoire in the tumor relative to less effective therapies

Total RNA was extracted from the tumor and spleen and reverse-transcribed into cDNA. TCRβ genes were amplified from cDNA and subjected to next generation sequencing. The TCR repertoire was evaluated based on the complementarity determining region 3 (CDR3) sequences. Although the numbers of total productive sequence counts were similar in tumors and spleens, the numbers of unique reads from the tumor were only about one-fifth of those of cells from spleens (Table 1). Consistently, Shannon diversity indices of the tumor in all groups of mice were smaller (<6.0) than those of the spleens (>9.0) regardless of treatment. These results indicated that T cell clones were selectively expanded in the tumor, probably because tumor-reactive T-cells were enriched. However, larger numbers of productive sequence counts were obtained from the tumors of treated groups than from untreated controls (Table 1). This is consistent with more specific TILs being present in the tumor of mice receiving these immunotherapies (Fig. 2). We also noted a significant increase in the number of productive unique TCRβ sequences in the tumors of treated mice (Table 1), suggesting that the emergence of large numbers of new T-cell clones resulted in a relatively increased richness of diversity of the TCR repertoire under treatment.

**Table 1:** TCR repertoire analysis of tumor and spleen in mice under immunotherapy.

		(–)	anti-PD-1	anti-CTLA-4	anti-4-1BB	anti-PD-1 + anti-4-1BB	anti-CD4
Tumor	No. of total reads/μg RNA (*2)	6.8 ± 2.8 × 10^3^	22.9 ± 11.8 × 10^3^ (*p* = 0.051)	22.7 ± 10.5 × 10^3^ (*p* = 0.051)	26.6 ± 18.1 × 10^3^ (*p* = 0.317)	99.1 ± 105.5 × 10^3^ (*p* = 0.051)	46.5 ± 34.0 × 10^3^ (*p* = 0.051)
	No. of unique reads (*1)	2.9 ± 0.8 × 10^2^	15.5 ± 7.4 × 10^2^ (*p* = 0.007)	16.2 ± 6.3 × 10^2^ (*p* = 0.004)	13.3 ± 9.1 × 10^2^ (*p* = 0.030)	12.1 ± 2.5 × 10^2^ (*p* = 0.064)	12.5 ± 2.1 × 10^2^ (*p* = 0.050)
	Shannon diversity index (*1)	4.1 ± 0.2	5.3 ± 0.9 (*p* = 0.006)	5.5 ± 0.4 (*p* = 0.002)	5.8 ± 0.5 (*p* < 0.001)	4.6 ± 0.6 (*p* = 0.462)	4.7 ± 0.4 (*p* = 0.224)
Spleen	No. of total reads/μg RNA (*1)	17.3 ± 0.7 × 10^3^	37.0 ± 12.5 × 10^3^ (*p* < 0.001)	23.5 ± 5.6 × 10^3^ (*p* = 0.507)	21.5 ± 6.3 × 10^3^ (*p* = 0.814)	20.9 ± 6.3 × 10^3^ (*p* = 0.890)	30.1 ± 4.7 × 10^3^ (*p* = 0.033)
	No. of unique reads (*1)	12.9 ± 1.5 × 10^3^	8.5 ± 1.4 × 10^3^ (*p* < 0.001)	9.6 ± 1.5 × 10^3^ (*p* = 0.014)	13.0 ± 2.7 × 10^3^ (*p* = *1.000*)	8.4 ± 1.2 × 10^3^ (*p* < 0.001)	6.3 ± 0.9 × 10^3^ (*p* < 0.001)
	Shannon diversity index (*1)	9.7 ± 0.2	9.1 ± 0.1 (*p* = 0.042)	9.3 ± 0.2 (*p* = 0.372)	9.1 ± 0.7 (*p* = 0.058)	8.2 ± 0.3 (*p* < 0.001)	8.8 ± 0.1 (*p* = 0.002)

Dunnett’s test (*1) or Steel’s test (*2) were used for multiple comparisons between control and treatment groups.

### Successful immunomodulatory therapies induce the enrichment of selected T-cell clones in the tumor

Some of the top 100 most frequent TCR sequences from the tumor were also detected in the spleen, but at low frequency (Supplementary Fig. S2). Depending on the TCR sequence, clonal enrichment was up to 6700-fold (but the mean enrichment was 96.2-fold). Immunotherapies applied in this study increased the number of these clonotypes shared between tumor and spleen. They were detected more frequently in mice receiving anti-4-1BB, the anti-PD-1/4-1BB combination or anti-CD4 mAb treatment than in mice on anti-PD-1 or anti-CTLA-4 monotherapy, or the untreated control. These results suggest that immunotherapies that can exert potent anti-tumor activity, such as anti-PD-1/4-1BB or anti-CD4 mAb in this study, successfully re-activate and expand putatively pre-existing tumor-reactive T-cells in the animal.

**Figure 5:**
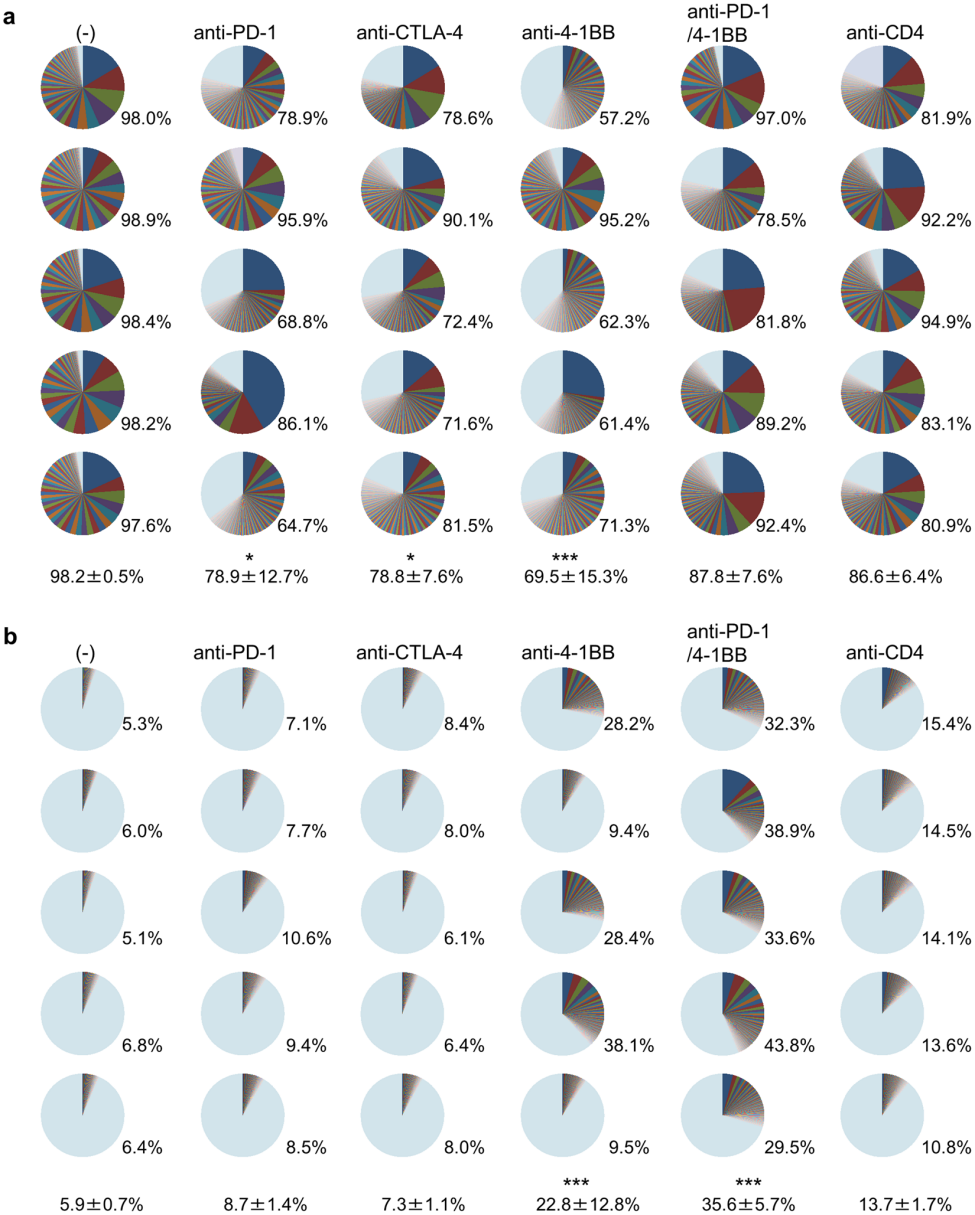
Percentage of the top 100 most frequency clones. TRB CDR3 clonotype diversity in the tumor (**a**) and the spleen (**b**) shown as pie charts for each of the 5 individual mice. The colors are automatically given by the software and do not correspond to identical TCR clonotypes. The number on the bottom at the right of each panel indicates the cumulative frequency of the top 100 clones of total productive read counts in individual mice. Mean ± SD of 5 mice per group is also indicated at the bottom. Dunnett’s test was used for multiple comparisons between control and treatment groups. *p < 0.05, ***p < 0.001.

The distribution of the percentage of the top 100 most frequent clones is illustrated for each of 5 mice as a pie chart in Fig. 5. The cumulative frequency of the top 100 clones constituted almost 100% of the total productive read counts in control mouse tumors, indicating selection for limited numbers of clonotypes. Nearly one-quarter or one-third of TILs in treatment groups consisted of less frequent clonotypes (Fig. 5a). These results again indicate an increased diversity of the T-cell repertoire in the tumor after immunotherapies, although the diversity of the TCR repertoire of T-cells in the tumor was much less than in the spleen (Fig. 5a and b).

**Figure 6:**
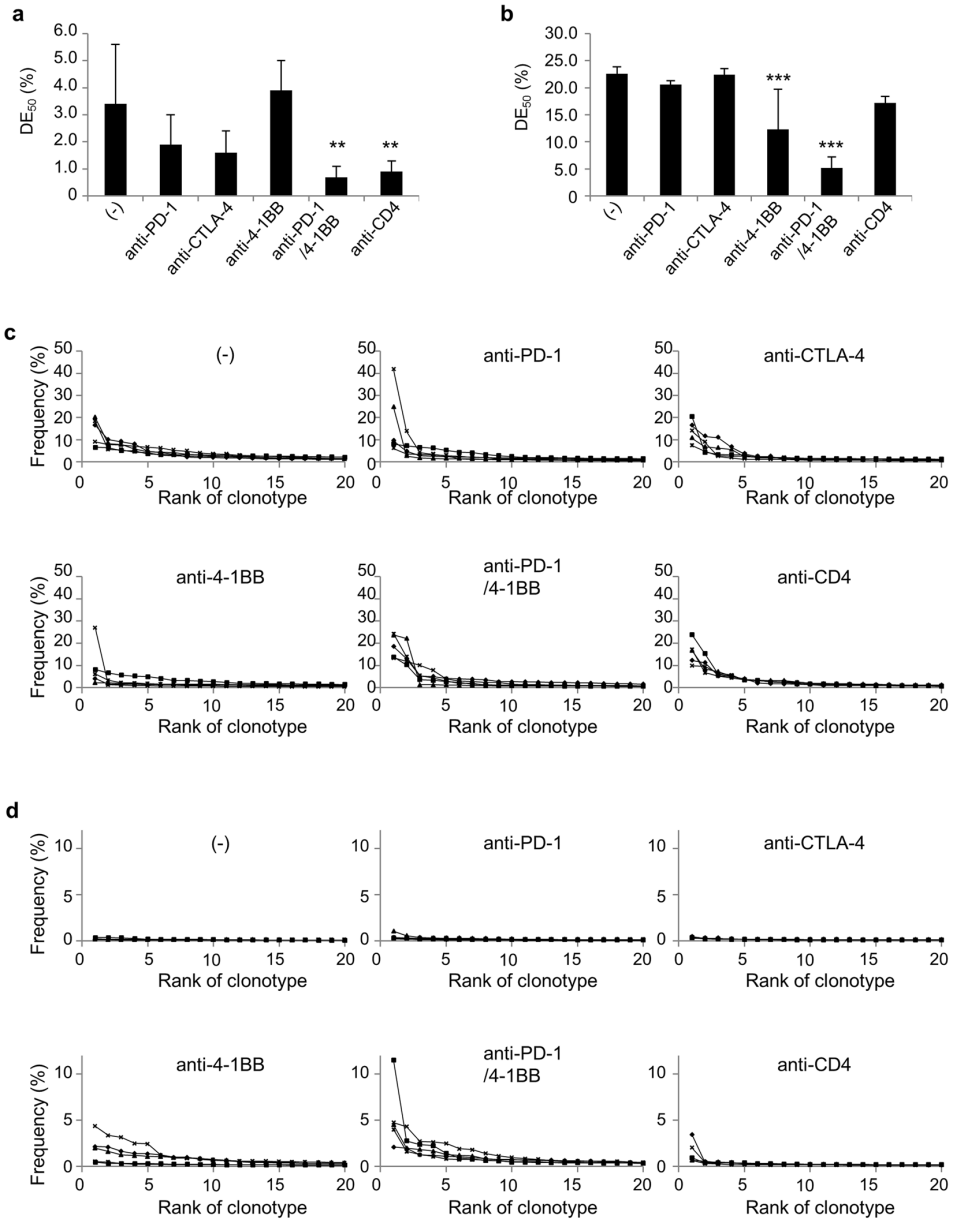
Evenness of TRB CDR3 clonotype distribution. Mice were treated as described in the legend to Fig. 4. Groups of mice (n = 5) were killed on day 14 and TCRβ sequencing was performed. Diversity Evenness 50 (DE_50_) scores of the tumor (**a**) and the spleen (**b**) in each indicated treatment were calculated by DE_50_ = (the number of unique reads that consist of 50% of total read count)/(the total unique read count). All unique CDR3 sequences detected in the tumor (**c**) and the spleen (**d**) in each indicated treatment group sorted according to their frequency within the sample, showing that larger clones (left) dominate in the tumor of mice receiving immunotherapies. In mice that received anti-4-1BB mAb, highly enriched T-cell clones were also detected in the spleen. Solid lines indicate each individual mouse. Dunnett’s test was used for multiple comparisons between control and treatment groups. **p < 0.01, ***p < 0.001.

Because diversity can be decomposed into richness and evenness, evenness values for TCR repertoire of the tumor were evaluated (Fig. 6). Diversity Evenness 50 (DE_50_) was calculated as the ratio of how many clonotypes amongst the most frequent were necessary to account for 50% of the total read counts divided by the total number of read counts present. High DE_50_ corresponds to each clonotype evenly represented in terms of frequency. Low evenness corresponds to a TCR repertoire dominated by certain specific CDR3s. DE_50_ of T-cells from the tumor (Fig. 6a) is lower than from spleens (Fig. 6b) in all experimental groups, even in control mice, suggesting that TILs were more oligoclonal than those of peripheral tissues. DE_50_ values of the tumor in anti-PD-1 or anti-CTLA-4 treated mice were lower than control mice. In contrast, DE_50_ of the tumor in anti-4-1BB monotherapy was larger than that of control mice, suggesting that more diverse T-cells were activated and infiltrated into the tumor. In the tumor of mice where the tumor growth was well-controlled by the combination of anti-PD-1/4-1BB or anti-CD4 treatment, DE_50_ values were significantly lower than those of control (Fig. 6a). There was no statistically significant difference in DE_50_ values between control and anti-CTLA-4 or anti-4-1BB mAb treated groups; no control of tumor growth was seen in those mice.

The frequency distribution of the top 20 unique TCR clonotypes is also depicted by sorting them according to their frequency within the sample (Fig. 6c and d). The x-axis represents each unique clonotype in descending order of frequency, and the frequency of read counts for each clonotype on the y-axis. In the tumor, expansion of dominant clones was observed, especially in mice receiving combination anti-PD-1/4-1BB, or anti-CD4 mAb treatment (Fig. 6c). These results indicate that the enrichment of selected T-cell clones in the tumor is essential for the successful immunotherapy.

### Effector function of enriched T-cells in the tumor and their anti-tumor activities

**Figure 7:**
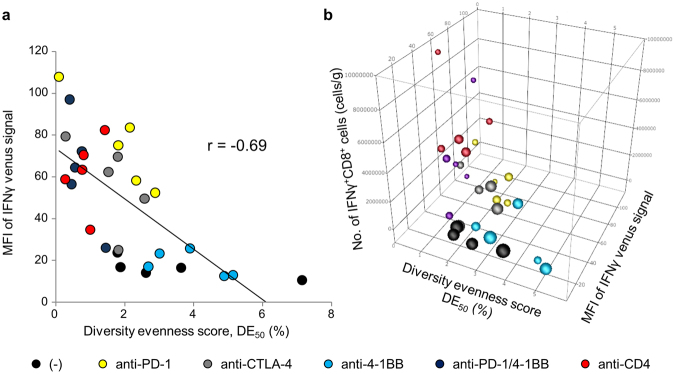
Correlation between TRB CDR3 clonotype diversity in the tumor, T-cell effector function and anti-tumor activities. (**a**) Diversity Evenness 50 (DE_50_) scores on the x-axis versus MFI values of IFNγ venus signal on the y-axis. Each dot indicates individual mice receiving no treatment (black circles), anti-PD-1(yellow), anti-CTLA-4 (grey), anti-4-1BB (blue), combination of anti-PD-1 and 4-1BB (purple) or anti-CD4 mAb (red). (**b**) Relationship between Diversity Evenness 50 (DE_50_) scores, MFI values of IFNγ venus signal, the number of IFNγ^+^CD8^+^ cells in the tumor (cells/gram) and the tumor volume. The size of the circle indicates the tumor volume.

TCR repertoire analysis revealed the expansion and enrichment of certain clonotypes in the tumor of mice receiving effective immunotherapy (Figs 5, 6 and Supplementary Fig. S2). Therefore, we assessed the correlation between these enriched T-cells and anti-tumor activity. As shown in Fig. 7a, the DE_50_ of the tumor was inversely correlated with the MFI of IFNγ venus signals of CD8^+^ T-cells, irrespective of the type of immunotherapy applied. MFI^high^ T-cells were mostly present in mice that received anti-PD-1, anti-PD-1/4-1BB or anti-CD4 and had a low DE_50_. T-cells in mice under anti-4-1BB monotherapy or untreated mice displayed higher DE_50_ and lower MFI than others. Collectively, our data indicate that tumor-reactive T-cell populations should be enriched in the tumor (low DE_50_), that they are fully functional as effectors (high MFI of IFNγ venus signals) and that it is the expansion of these T-cells which makes the immunotherapy effective (Fig. 7b).

### Polyclonal T-cell activation in the periphery is associated with immune-related adverse events

T-cells activated by the immunotherapies applied in this study are not expected to be antigen-specific and are not necessarily directed to tumor antigens. T-cells reactive to tissues may also be activated as well. We observed that IFNγ venus^+^ T-cells were increased in the spleens of all mice (Fig. 3 and Supplementary Fig. S1) with the expansion of many clonotypes in mice receiving immunotherapies (Figs 5 and 6). Emergence of dominant clones in the spleen was particularly noticeable in mice that received anti-4-1BB, the anti-PD-1/4-1BB combination or anti-CD4 mAb (Figs 5b and 6d). DE_50_ values of the spleens in these mice were lower than in the others (Fig. 6b). Therefore, we performed a histological analysis of various organs from these mice (Supplementary Fig. S3). Infiltration of mononuclear cells around the portal area was observed in the liver of mice receiving anti-4-1BB mAb; mononuclear cell infiltration into the liver parenchyma was additionally seen under anti-PD-1/4-1BB mAb treatment (Supplementary Fig. S3b). Kidney, heart, lung and pancreas were also involved (Supplementary Fig. S3d,f,h,j). Mononuclear cell infiltrations around the renal tubules, sub-pericardium of the heart, interstitial space of the lung, islet and pancreatic duct (Supplementary Fig. S3d,f,h,j). Liver damage was confirmed by the elevated serum ALT levels in these mice (Supplementary Fig. S3k). Serum glucose levels were within the normal range (Supplementary Fig. S3l). Consistent with the level of T-cell activation, mononuclear cell infiltrations into organs were severe in mice receiving anti-4-1BB and anti-PD-1/4-1BB combination therapy (Supplementary Fig. S4). Surprisingly, mononuclear cell infiltrations were scarce in the anti-CD4 mAb group, even though activation and expansion of T-cells was detected in the spleens (Figs 5, 6 and Supplementary Fig. S4). This is probably because the depletion of CD4^+^ T-cells eliminated the autoreactive cells.

## Discussion

To increase the efficacy of immunotherapy, strategies to activate the immune system need to be combined with prevention of immune suppression^8,9^. It has been reported that robust and sustained anti-tumor effects are more frequently observed with a combination of anti–CTLA-4 and anti–PD-1 mAbs than with each agent alone^10^. Consistent with this, the most marked anti-tumor activity in the present study was observed using a combination of anit-PD-1/4-1BB mAb (Fig. 1). It remains to be determined how the most effective therapeutic combinations can be developed. To this end, we focused on the characteristics of the intratumoral immune responses induced by several different immunotherapies that did or did not confer clinical benefit in a mouse model, as measured by suppression of tumor growth. T-cell activation was demonstrated by their production of IFNγ assessed by venus signals in this model. Expansion of antigen-specific T-cells was monitored using gp100-specific TCR-transgenic pmel-1 cells. According to this readout, all of the immunotherapies applied in this study succeeded in activating and inducing the expansion of T-cells in the tumor to a greater or lesser extent. Nonetheless, suppression of tumor growth was achieved only by anti-PD-1, or the combination of anti-PD-1 with the agonistic antibody anti-4-1BB, as well as by anti-CD4 mAb monotherapy. In contrast, the tumor grew progressively in mice treated with anti-CTLA-4 or anti-4-1BB mAb monotherapy in this challenging B16 melanoma model. The absolute number of CD8^+^ T-cells in the tumor, their effector function, and the richness and evenness of TCR repertoire diversity in the tumor were found to be closely associated with whether the immunotherapy was successful or not.

To obtain full activation of CD8^+^ T-cells in the tumor, strategies to overcome the local immunosuppressive microenvironment are required^8^. Even though tumor-reactive CD8^+^ T-cells are activated, they may be counter-regulated by several mechanisms referred to as adaptive or acquired resistance^11,12^. In the present study, the majority of CD8^+^ T-cells in the tumor were also venus^+^, suggesting that they were reactive to the tumor (Fig. 3). However, even though such CD8^+^ T-cells were present in the tumor of untreated mice or when expansion of these CD8^+^ T-cells was induced by treatment with the immunostimulatory anti-4-1BB mAb, their expression of venus signals was weak and control of tumor growth was not achieved in these mice (Fig. 1). Nonetheless, although venus^high^ CD8^+^ T-cells were detected in mice receiving anti-PD-1, anti-CTLA-4, the anti-PD-1/4-1BB combination or anti-CD4 mAbs (Fig. 3), tumor growth was still not controlled in the anti-CTLA-4 group (Fig. 1). These results indicate that the exertion of full effector function by tumor-infiltrating CD8^+^ T-cells under immunomodulatory therapy is necessary but not in itself sufficient for tumor growth suppression.

The diversity of the TCR repertoire, reflecting clonal composition, the potential spectrum of antigen recognition and the quantity of available T-cells responding, rather than the mere presence of some responding cells, may be the crucial factor responsible for these differences. TCR repertoire diversity can be quantified by next-generation TCR sequencing (Figs 5–7 and Supplementary Fig. S2). It has been reported that Sipuleucel-T treatment increases TIL diversity in prostate tumors^13^ and that CTLA-4 blockade promotes active remodeling of the T-cell repertoire leading to increased repertoire diversity overall^14,15^. However, in a clinical trial of anti-PD-1 mAb therapy, expansion of certain clones resulted in a less diverse T-cell population^16^. In our study, Shannon diversity indices were not correlated with anti-tumor activity (Table 1). Although TCR diversity could explain tumor-associated immune responses, the relationship between T-cell diversity and the anti-tumor immune response is still being explored. Therefore, we decomposed T-cell diversity into richness (the number of unique elements in a population) and evenness (the distribution of the frequencies of those elements) (Fig. 6). The concept of evenness is more usually applied in ecology^17^. Because the richness cannot discriminate the samples that contain very few expanded clones with several rare ones, or those evenly distributed ones, discriminating between these contrasting clonal compositions by evenness score is useful. Recently, Riaz *et al*. reported that decreased evenness without a significant change in the total number of CDR3s (richness) in patients received nivolumab^18^. The richness was increased by all immunotherapies in this study irrespective of their beneficial effect, whereas our results for evenness indicate that effective treatment by anti-PD-1, a combination of anti-PD-1 and 4-1BB, or anti-CD4 monotherapy, increases the expansion of selected clones in the tumor resulting in less evenness in the T-cell population (Fig. 6). Here, we have used DE_50_ values to describe TCR repertoire analysis and find good correlations with the strength of the IFNγ venus signals we measured as an indicator of effector function of CD8^+^ T-cells in the tumor (Fig. 7a).

Recently, Huang *et al*. reported that the degree of reinvigoration of effector T-cells by anti-PD-1 mAb depended on the initial tumor burden and was critical for effective therapy^19^. Consistent with this, we observed that higher numbers of all CD8^+^ T-cells (Fig. 2) and of venus^+^ CD8^+^ T-cells (Fig. 3) in the tumor were well-associated with the suppression of tumor growth resulting from anti-PD-1/4-1BB or anti-CD4 mAb therapy. Collectively, our results indicate that effective immunotherapies in this model meet the following criteria (Fig. 7): low DE_50_, high MFI of IFNγ venus signals in CD8^+^ T-cells and the presence of a large number of IFNγ^+^ CD8^+^ T-cells in the tumor. First, low DE_50_ indicates the selective expansion of tumor-reactive T-cell populations in the tumor. Second, the presence of venus^hi^ CD8^+^ T-cells marks the reinvigoration of T-cells with full effector function. Third, the magnitude of these effector T-cell expansions overcomes the negative effect of high tumor burden.

Finally, one of the major concerns with checkpoint blockade is the occurrence of immune-related adverse events^20^, because immunotherapies that lack antigen-specificity have the potential to activate and expand autoreactive T-cell populations as well as tumor-reactive T-cells. In our study, mononuclear cell infiltration into the liver, kidney, heart, lung and pancreas was observed more frequently in mice that received anti-4-1BB mAb monotherapy and combination with anti-PD-1 mAb (Supplementary Fig. S3). These autoimmune responses were associated with the expansion of certain T-cell clonotypes in the spleen (Figs 5b and 6d) and a lower DE_50_ in the spleen of these animals (Fig. 6b). These results are consistent with a previous report that immune toxicities are associated with an increased diversity of the T-cell repertoire under anti-CTLA-4 treatment^21^. TCR repertoire analysis and evaluation of diversity are useful biomarkers to assess immune-related adverse events of immunotherapies.

In conclusion, TCR repertoire analysis of T-cells in the tumor and periphery is a valuable tool for guiding the development of effective immunotherapies avoiding immune-related adverse events. Selective expansion of fully functional tumor-reactive T-cells is reflected in increased diversity that can be decomposed into increased richness and reduced evenness of the T-cell repertoire. For successful immunotherapy, the optimal combination therapy is the one that increases T-cell diversity in the tumor without affecting the peripheral repertoire.

## Methods

### Mice and cell lines

Female C57BL/6 mice at the age of 6–8 wk were obtained from Japan SLC (Sizuoka, Japan). Pmel-1-TCR transgenic mice recognizing the H-2D^b^-restricted epitope EGSRNQDWL from gp100 (gp100 25–33) were obtained from The Jackson Laboratory (Bar Harbor, ME)^22^. The strain is also homozygous for the T lymphocyte specific Thy1a (Thy1.1) allele. IFNγ Venus reporter mice were described previously^23^. All mice were kept in a specific pathogen-free environment. The animal use proposal and experimental protocols have been reviewed and approved by the University of Tokyo Animal Care and Use Committee (ID: P15-122) and all animal procedures were conducted in accordance with institutional guidelines. B16F10 is a gp100^+^ spontaneous murine melanoma cell line, kindly provided by Dr. N. Restifo (National Cancer Institute, MD). B16F10 cells were transduced with the truncated form of human low-affinity nerve growth factor receptor (ΔhLNGFR/hCD271)^7^. These cells were maintained in culture medium consisting of DMEM (Wako Pure Chemical, Osaka, Japan) with 10% heat-inactivated fetal bovine serum (SAFC Biosciences, Lenexa, KS, USA), 100 μg/ml streptomycin and 100 U/ml penicillin (Wako Pure Chemical).

### Tumor models

C57BL/6 mice or IFNγ venus mice were inoculated with 5 × 10^5^ ΔhLNGFR/B16F10 cells subcutaneously. To allow us to directly visualize antigen-specific CD8^+^ T-cell responses *ex vivo*, the frequency of gp100-specific CTL precursors was increased by intravenously injection of 5.0 × 10^4^ CD90.1^+^CD8^+^ pmel-1 TCR Tg T-cells the day before tumor inoculation. On days 5 and 9 after tumor inoculation, mice received intraperitoneal injections of 200 μg anti-PD-1 (clone RMP1-14), anti-CTLA-4 (clone 9H10), anti-4-1BB (clone 3H3), anti-CD4 (clone GK1.5) mAbs or a combination of anti-PD-1 and anti-4-1BB mAb. Anti-4-1BB mAb was kindly provided by Dr. Mittler (Emory Vaccine Center, GA); other mAbs for *in vivo* use were purchased from BioXcell (West Labanon, NH, USA). Tumor growth was monitored every 2 to 3 days with calipers in a blinded fashion and was performed independently at least twice with similar results. Tumor volume was calculated by the formula π/6 × L_1_L_2_H, where L_1_ is the long diameter, L_2_ is the short diameter, and H is the height of the tumor.

### Cell preparation and flow cytometry

Tumor-infiltrating cells were prepared using a tumor dissociation kit (Miltenyi Biotec Inc., Auburn, CA, USA) according to the manufacturer’s instructions. Briefly, tumors were harvested from mice at the indicated time points, cut into pieces, and transferred to gentle-MACS C Tubes containing an enzyme mix (Miltenyi) and passed through a 70 μm cell strainer (Fisher Scientific, Hampton, NH) to obtain tumor-infiltrating cells. Cells from draining LNs, non-draining LNs, and spleens of each group (5 mice) were pooled and analyzed. To eliminate dead cells, the preparations were stained with Zombie Yellow (BioLegend, San Diego, CA). The cells were then pretreated with Fc Block (anti-CD16/32 clone 2.4G2; BioXcell), stained with antibodies and analyzed on a Gallios™ flow cytometer (Beckman-Coulter, Brea, CA). The following mAbs were obtained from BioLegend and used for flow cytometry: PE-conjugated anti-CD4, anti-PD-L1, PerCP/Cy5.5-conjugated anti-CD45, anti-LNGFR, AlexaFluor 647-conjugated anti-CD90.1, Alexa Fluor 700-conjugated anti-CD3, pacific blue-conjugated anti-CD8. Data were analyzed with FlowJo software (version 10; FlowJo LLC, Ashland, OR). Total numbers of cells were estimated from a FACS-based cell count of single-cell suspensions. Flowcount beads (Beckman-Coulter) were added to the cell samples and cell counts were calculated by the following equation: viable cells × total beads/counted beads.

### TCR repertoire analysis

Tissues were homogenized in TRIzol (Ambion, Carlsbad, CA). RNA was extracted from each sample using the RNeasy mini kit (QIAGEN, Hilden, Germany) and amounts and purity measured with the Agilent 2200 TapeStation (Agilent Technologies, Palo Alto). Total RNA was converted into complementary DNA (cDNA) with Superscript III reverse transcriptase (Invitrogen, Carlsbad, CA). Next, TCR genes were amplified using adaptor ligation-mediated PCR^24^. High-throughput sequencing was performed using the Illumina Miseq paired-end platform (2 × 300 bp) (Illumina, San Diego, CA). Assignment of TRBV and TRBJ segments in TCR genes was performed based on the international ImMunoGeneTics information system® (IMGT) database (http://www.imgt.org). Data processing, assignment, and data aggregation were automatically performed using repertoire analysis software originally developed by our group (Repertoire Genesis, Osaka, Japan). A unique sequence read was defined as a sequence read having no identity in TRBV, TRBJ and deduced amino acid sequence of CDR3 with the other sequence reads. The copy number of identical unique sequence reads was automatically counted by RG software in each sample and then ranked in order of the copy number. Total read counts were adjusted by the amount of input mRNA (read count/μg). Percentage occurrence frequencies of sequence reads with TRBV and TRBJ genes in total sequence reads were calculated.

### Histology

Tissues were fixed in 10% neutral formalin (Muto Pure Chemicals, Tokyo, Japan), embedded in paraffin, sectioned (3 mm), and stained with hematoxylin and eosin. Images were obtained using BZ-9000 microscope (KEYENCE, Osaka, Japan).

### Biochemical test

The increase in serum alanine transferase (ALT) concentration, which is an indicator of liver damage, was measured on a Fuji DRY-CHEM 5500 V (Fuji Medical Systems, Tokyo, Japan)^25^. The serum glucose concentration was measured by HITACHI 7180 biochemistry automatic analyzer (HITACHI, Tokyo, Japan).

### Statistical analysis

Statistical analyses were performed with JMP software, version 11.0.0. (SAS Institute Inc., Cary, NC). Results are shown as mean ± SD. Dunnett’s test or Steel’s test were used for multiple comparisons between a control group and treatment groups.

## Electronic supplementary material

Supplementary Figures

## References

[1] Hodi FS (2010). Improved survival with ipilimumab in patients with metastatic melanoma. The New England journal of medicine.

[2] Topalian SL (2012). Safety, activity, and immune correlates of anti-PD-1 antibody in cancer. The New England journal of medicine.

[3] Sharma P, Allison JP (2015). The future of immune checkpoint therapy. Science (New York, N.Y.).

[4] Callahan MK, Postow MA, Wolchok JD (2016). Targeting T Cell Co-receptors for Cancer Therapy. Immunity.

[5] Topalian SL, Drake CG, Pardoll DM (2015). Immune checkpoint blockade: a common denominator approach to cancer therapy. Cancer cell.

[6] Sun Y (2002). Costimulatory molecule-targeted antibody therapy of a spontaneous autoimmune disease. Nature medicine.

[7] Ueha S (2015). Robust Antitumor Effects of Combined Anti-CD4-Depleting Antibody and Anti-PD-1/PD-L1 Immune Checkpoint Antibody Treatment in Mice. Cancer immunology research.

[8] Pardoll DM (2012). The blockade of immune checkpoints in cancer immunotherapy. Nature reviews. Cancer.

[9] Duraiswamy J, Kaluza KM, Freeman GJ, Coukos G (2013). Dual blockade of PD-1 and CTLA-4 combined with tumor vaccine effectively restores T-cell rejection function in tumors. Cancer research.

[10] Wolchok JD (2013). Nivolumab plus ipilimumab in advanced melanoma. The New England journal of medicine.

[11] Restifo NP, Smyth MJ, Snyder A (2016). Acquired resistance to immunotherapy and future challenges. Nature reviews. Cancer.

[12] Sharma P, Hu-Lieskovan S, Wargo JA, Ribas A (2017). Primary, Adaptive, and Acquired Resistance to Cancer Immunotherapy. Cell.

[13] Sheikh N (2016). Clonotypic Diversification of Intratumoral T Cells Following Sipuleucel-T Treatment in Prostate Cancer Subjects. Cancer research.

[14] Cha E (2014). Improved survival with T cell clonotype stability after anti-CTLA-4 treatment in cancer patients. Science translational medicine.

[15] Robert L (2014). CTLA4 blockade broadens the peripheral T-cell receptor repertoire. Clinical cancer research: an official journal of the American Association for Cancer Research.

[16] Tumeh PC (2014). PD-1 blockade induces responses by inhibiting adaptive immune resistance. Nature.

[17] Arnaud-Haond S, Duarte CM, Alberto F, Serrao EA (2007). Standardizing methods to address clonality in population studies. Molecular ecology.

[18] Riaz N (2017). Tumor and Microenvironment Evolution during Immunotherapy with Nivolumab. Cell.

[19] Huang AC (2017). T-cell invigoration to tumour burden ratio associated with anti-PD-1 response. Nature.

[20] De Velasco G (2017). Comprehensive Meta-analysis of Key Immune-Related Adverse Events from CTLA-4 and PD-1/PD-L1 Inhibitors in Cancer Patients. Cancer immunology research.

[21] Oh DY (2017). Immune Toxicities Elicted by CTLA-4 Blockade in Cancer Patients Are Associated with Early Diversification of the T-cell Repertoire. Cancer research.

[22] Overwijk WW (2003). Tumor regression and autoimmunity after reversal of a functionally tolerant state of self-reactive CD8 + T cells. The Journal of experimental medicine.

[23] Miyauchi K (2016). Protective neutralizing influenza antibody response in the absence of T follicular helper cells. Nat Immunol.

[24] Kitaura K, Shini T, Matsutani T, Suzuki R (2016). A new high-throughput sequencing method for determining diversity and similarity of T cell receptor (TCR) alpha and beta repertoires and identifying potential new invariant TCR alpha chains. BMC immunology.

[25] Ueha S (2007). Intervention of MAdCAM-1 or fractalkine alleviates graft-versus-host reaction associated intestinal injury while preserving graft-versus-tumor effects. J Leukoc Biol.

