# Author Correction: Increased diversity with reduced “diversity evenness” of tumor infiltrating T-cells for the successful cancer immunotherapy

**DOI:** 10.1038/s41598-023-33836-2

**Published:** 2023-04-26

**Authors:** Akihiro Hosoi, Kazuyoshi Takeda, Koji Nagaoka, Tamaki Iino, Hirokazu Matsushita, Satoshi Ueha, Shin Aoki, Kouji Matsushima, Masato Kubo, Teppei Morikawa, Kazutaka Kitaura, Ryuji Suzuki, Kazuhiro Kakimi

**Affiliations:** 1 Department of Immunotherapeutics, The University of Tokyo Hospital, 7-3-1 Hongo, Bunkyo-ku, Tokyo, 113-8655 Japan; 2 Division of Cell Biology, Biomedical Research Center, Graduate School of Medicine, Juntendo University, Bunkyo-ku, Tokyo, 113-8421 Japan; 3 Department of Biofunctional Microbiota, Graduate School of Medicine, Juntendo University, Bunkyo-ku, Tokyo, 113-8421 Japan; 4 Department of Molecular Preventive Medicine, Graduate School of Medicine, The University of Tokyo, 7-3-1 Hongo, Bunkyo-ku, Tokyo, 113-0033 Japan; 5 Laboratory for Cytokine Regulation, Research Center for Integrative Medical Science (IMS), RIKEN Yokohama Institute, Yokohama, Kanagawa 230-0045 Japan; 6 Division of Molecular Pathology, Research Institute for Biomedical Science, Tokyo University of Science, Noda, Chiba 278-0022 Japan; 7 Department of Pathology, Graduate School of Medicine, The University of Tokyo, 7-3-1 Hongo, Bunkyo-ku, Tokyo, 113-0033 Japan; 8 Repertoire Genesis Inc., Saito Bioincubator 104, 7-7-15 Saito-Asagi, Ibaraki-shi, Osaka, 567-0085 Japan; 9 MEDINET Co. Ltd,, 2-3-12 Shin-Yokohama, Kohoku-ku, Yokohama, Kanagawa 222-0033 Japan

Correction to: *Scientific Reports* 10.1038/s41598-018-19548-y, published online 18 January 2018

This Article contains an error in the Results section, under subheading ‘Successful immunomodulatory therapies induce the enrichment of selected T-cell clones in the tumor’,

“Diversity Evenness 50 (DE_50_) was calculated as the ratio of how many clonotypes amongst the most frequent were necessary to account for 50% of the total read counts divided by the total number of read counts present.”

should read:

“Diversity Evenness 50 (DE_50_) was calculated as the ratio of how many clonotypes amongst the most frequent were necessary to account for 50% of the total read counts divided by the total number of clonotypes present.”

