# Supplementary material for: Increased diversity with reduced “diversity evenness” of tumor infiltrating T-cells for the successful cancer immunotherapy
Source: Sci Rep. 2018 Jan 18;8:1058. doi: 10.1038/s41598-018-19548-y (PMC5773695; doi:10.1038/s41598-018-19548-y)
Supplement: Supplementary file 1 — Supplementary Figures [file 41598_2018_19548_MOESM1_ESM.pdf]

Supplementary Information

Increased diversity with reduced “diversity evenness” of tumor infiltrating T-cells for the successful cancer immunotherapy

Akihiro Hosoi, Kazuyoshi Takeda, Koji Nagaoka, Tamaki Iino, Hirokazu Matsushita, Satoshi Ueha, Shin Aoki, Kouji Matsushima, Masato Kubo, Teppei Morikawa, Kazutaka Kitaura, Ryuji Suzuki and Kazuhiro Kakimi

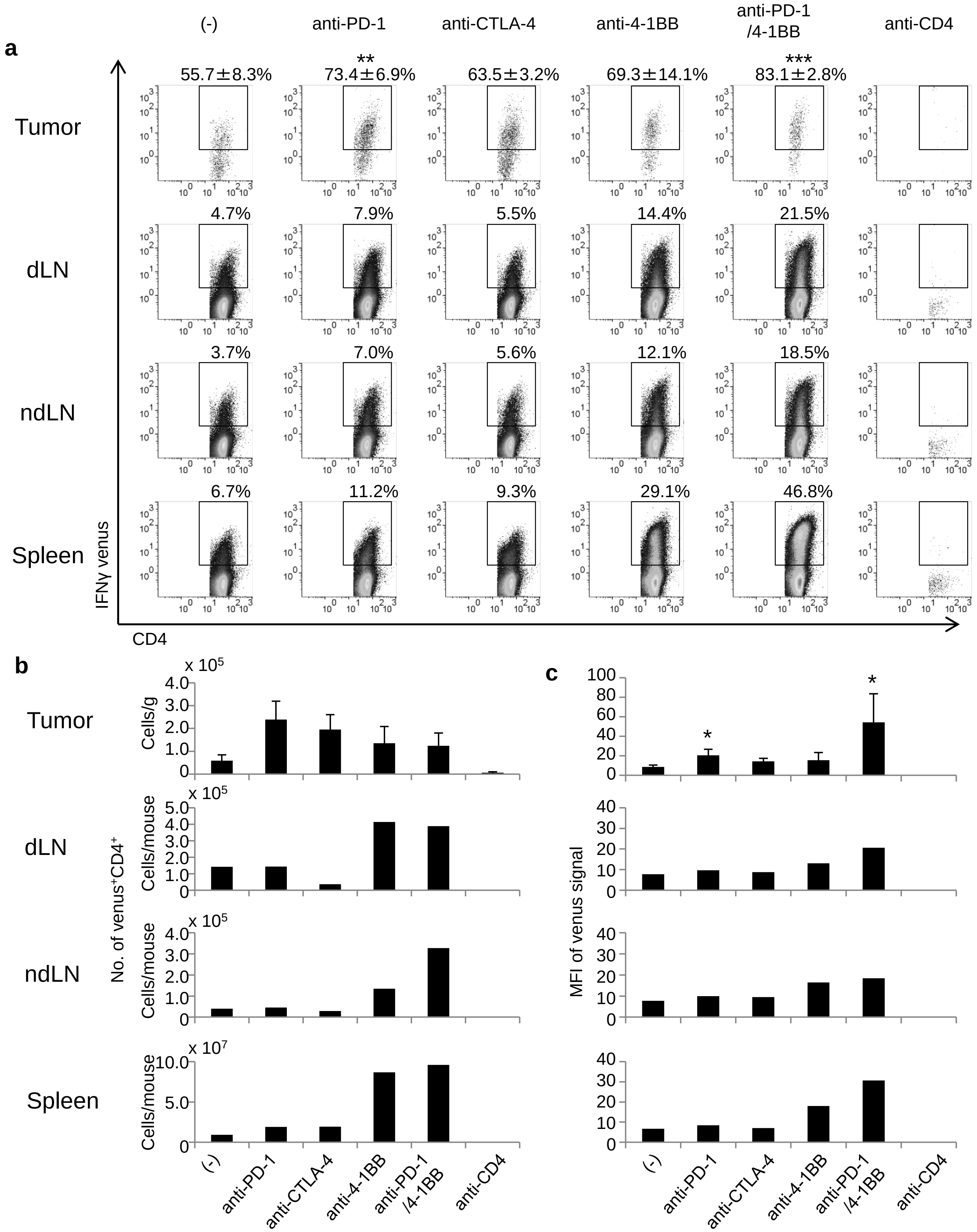

Supplementary Figure S1. Activation of CD4+ T-cells by immunotherapies.

(a) Mice were treated as described in the legend to Fig. 1. Mice (n=5) were killed on day 14 and IFNγ venus signals from CD4+ T-cells in the tumor, draining lymph node (dLN), non-draining lymph node (ndLN) and spleen were analyzed by flow cytometry. (b) The absolute numbers of venus+ CD4+ T-cells and (c) mean fluorescent intensities (MFI) of venus signals of these cells were compared. *p<0.05

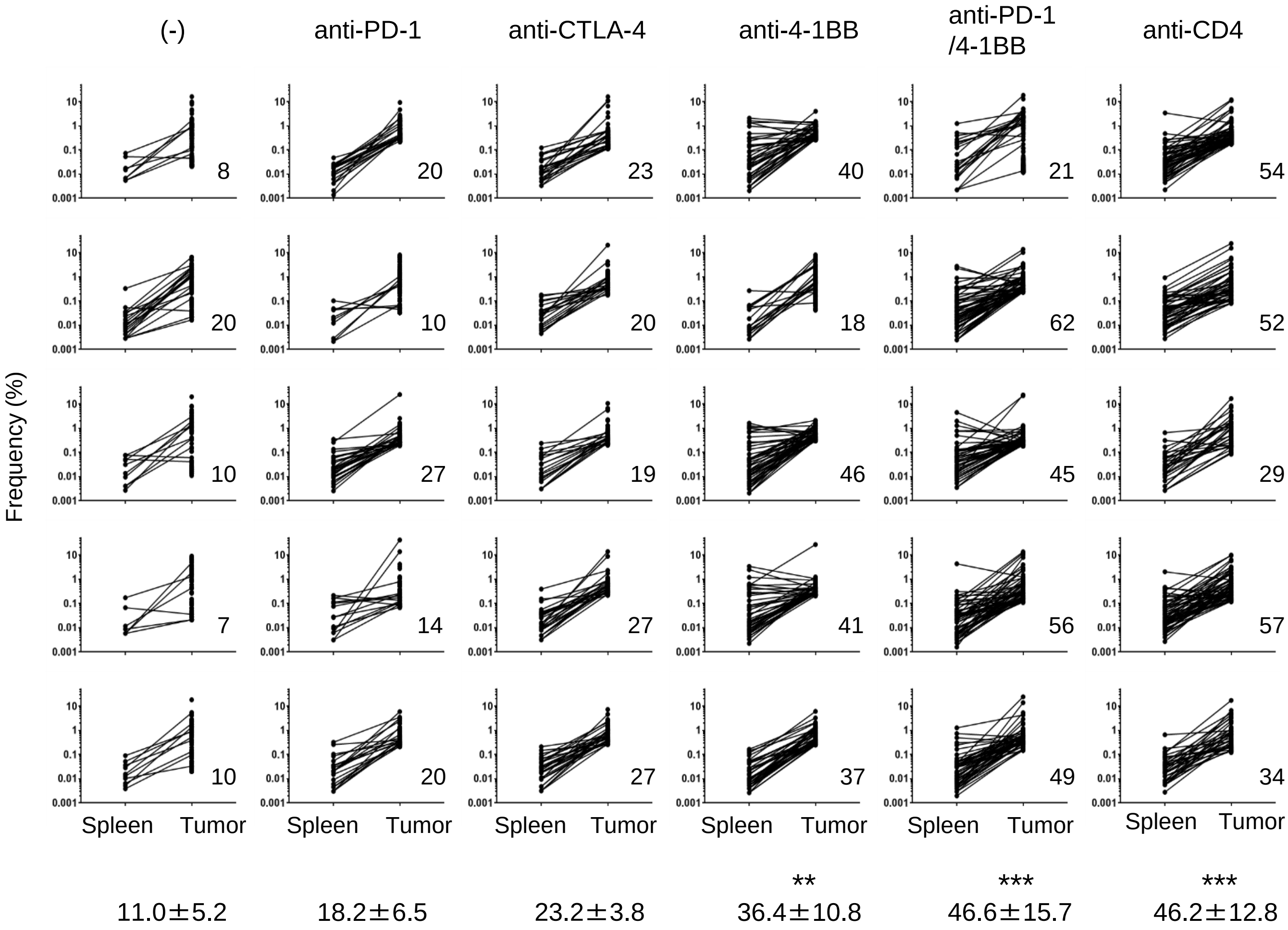

Supplementary Figure S2. Size of the top 100 most frequent TCRβ sequences detected in the tumor vs. spleen.

Mice were treated as described in the legend to Fig. 4. Groups of mice (n=5) were killed on day 14 and TCRβ sequencing was performed. Each panel depict the top 100 clones in the tumor and identical clones shared in the spleen of individual mice. The numbers on the bottom at the right indicate the numbers of clonotypes shared between tumor and spleen. Mean ± SD of each group of 5 mice is also indicated at the bottom. Dunnett's test was used for multiple comparisons between control and treatment groups. ** $p<0.01$, *** $p<0.001$

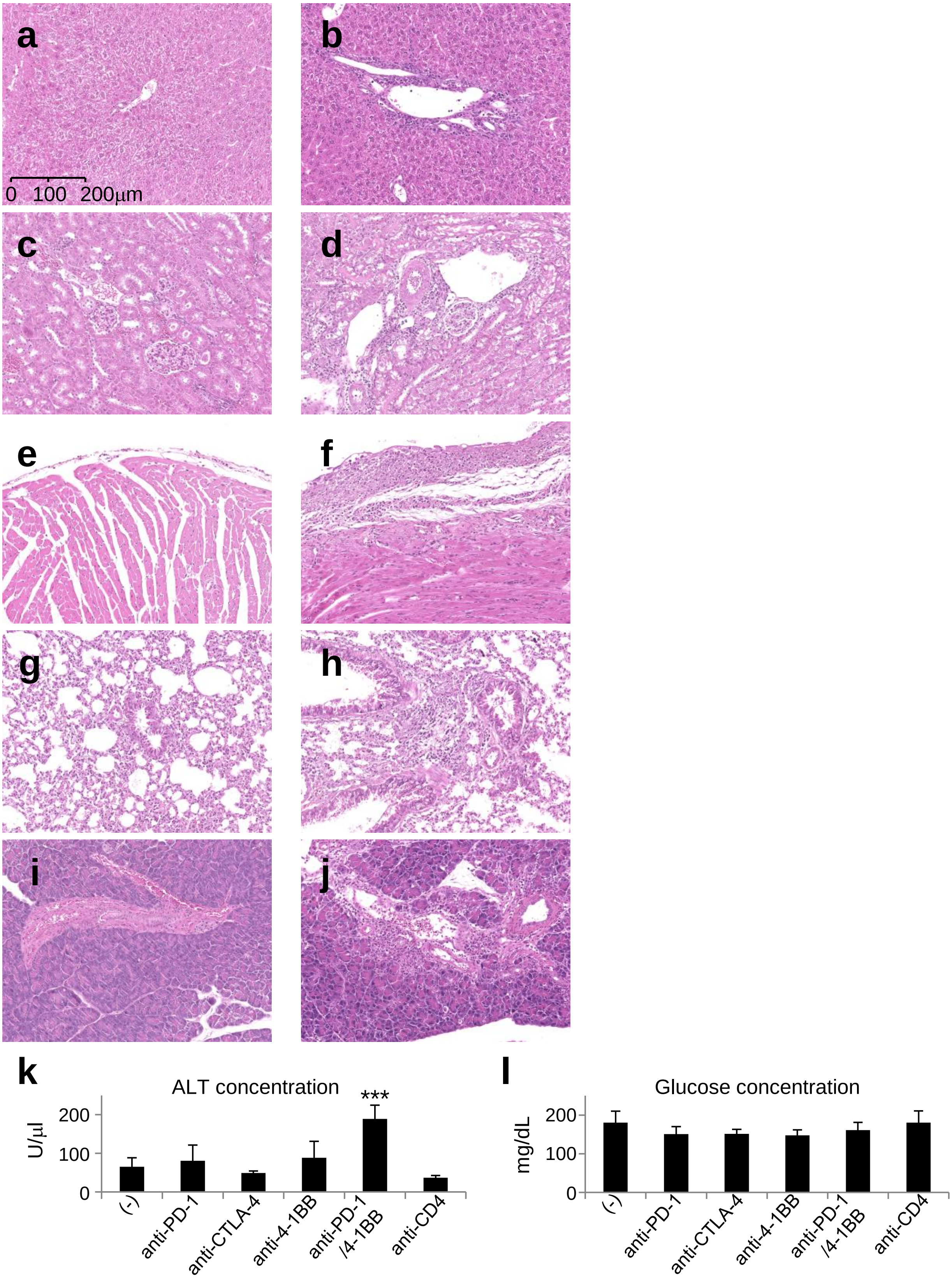

Supplementary Figure S3. Immune-related adverse events induced by immunotherapies.
Mice were treated as described in the legend to Fig. 4. Groups of mice (n=5) were killed on day 14. Liver (a, b), kidney (c, d), heart (e, f), lung (g, h) and pancreas (i, j) were harvested, formalin-fixed and embedded in paraffin. Panels a, c, e, g and i were from control mice; panels b, d, f, h and j were from anti-PD-1/4-1BB combination group. Sections were stained with hematoxylin and eosin. Scale bars, 200 μm. Serum ALT value (k) and glucose level (l) at the time of sacrifice were indicated. Bar graph indicates mean ± SD of 5 mice per group. ****p*<0.001

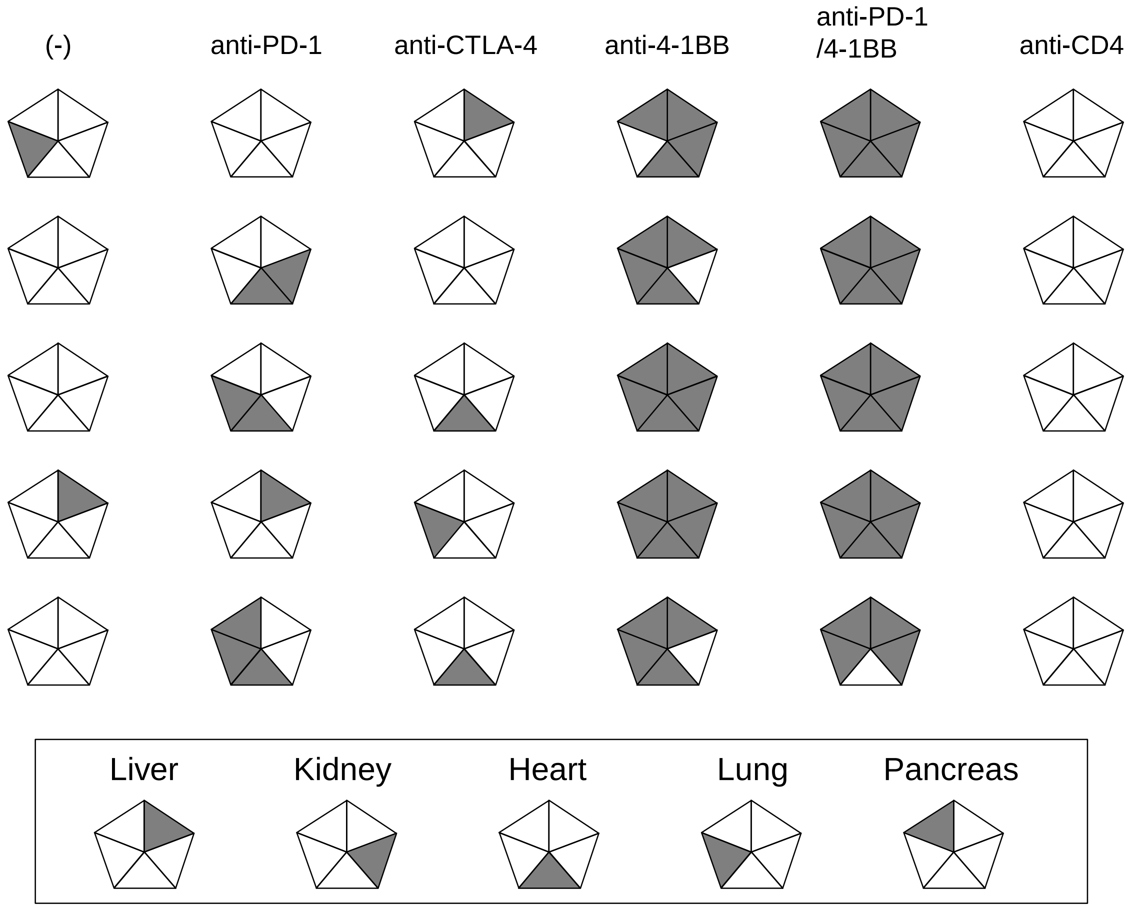

Supplementary Figure S4. Schematic of inflammatory cell infiltration into the peripheral tissues. Each pentagon represents a single mouse (n = 5). The gray triangle indicates the detection of inflammatory cell infiltration in the organ.
